# Australian aquatic bio-optical dataset with applications for satellite calibration, algorithm development and validation

**DOI:** 10.1016/j.dib.2022.108489

**Published:** 2022-07-26

**Authors:** Nathan Drayson, Janet Anstee, Hannelie Botha, Gemma Kerrisk, Phillip Ford, Bozena Wojtasiewicz, Lesley Clementson, James McLaughlin, Marlee Hutton

**Affiliations:** a CSIRO, Oceans and Atmosphere, Black Mountain, Canberra, ACT 2601, Australia; b CSIRO, Oceans and Atmosphere, Castray Esplanade, Battery Point TAS 7004, Australia; c CSIRO, Oceans and Atmosphere, Crawley, WA 6009, Australia

**Keywords:** Aquatic bio-optical properties, Inland water quality, Aquatic remote sensing reflectance, Aquatic remote sensing algorithm development

## Abstract

The authors present bio-optical data spanning 316 sets of observations made at 34 inland waterbodies in Australia. The data was collected over the period 2013–2021 and comprise radiometric measurements of remote sensing reflectance (Rrs), diffuse attenuation extinction coefficient (Kd); optical backscattering; absorption of coloured dissolved organic matter (aCDOM), phytoplankton (aph) and non-algal particles (aNAP); HPLC analysis of algal pigments including chlorophyll-a (CHL-a); organic and inorganic total suspended solids (TSS); and total and dissolved organic carbon concentration. Data collection has been timed to coincide with either Landsat 8 or Sentinel-2 overpasses. The dataset covers a diverse range of optical water types and is suitable for algorithm development, satellite calibration and validation as well as machine learning applications.

## Specifications Table

**Table:** 

Subject	Hydrology and Water quality
Specific subject area	Earth observation water quality
Type of data	Table
How the data were acquired	In situ instruments: Backscattering - WetLabs BB9, Wetlabs ECOTriplet BB2; Radiometry – RAMSES ACC-2 VIS planar irradiance sensor, RAMSES ACC-2 diffuse irradiance sensor, RAMSES ARC VIS radiance sensor Laboratory analysis of water samples: Absorption coefficients - dual-beam spectrophotometer (GBC Scientific Equipment Ltd., Cintra 404); software: Cintral ver. 2.2); Pigments – HPLC method described in [1] TSS – gravimetric determination on pre-rinsed, pre-weighed glass fibre filters. Total and dissolved organic carbon – Shimadzu Total Organic Carbon analyser after acidification and purging with high purity nitrogen.
Data format	Raw, analysed
Description of data collection	Data was collected using in situ instrumentation and laboratory analysis of water samples.
Data source location	Institution: CSIRO City/Town/Region: ACT Country: Australia
Data accessibility	Repository name: CSIRO Data Access Portal Data identification number: https://doi.org/10.25919/rtd7-j815 Direct URL to data: https://data.csiro.au/collection/csiro:54602v1

## Value of the Data

• Unique description of a broad range of bio-optical properties for Australian inland waters.
• Basis for parameterisation and training for semi-analytical and machine learning inversion algorithms.
• Validation dataset for inversion algorithms.
• Examining relationships between inherent optical properties to validate machine learning training datasets.
• Determine relationship between apparent and inherent optical properties of lakes.

## 1 Data Description

Monitoring water pollution using remote sensing offers a greater understanding of spatial and temporal distribution of pollutants than traditional methods of *in situ* data collection [2,3]. The calibration and validation of models used to predict concentrations of optically active constituents (OACs) from remotely sensed data require representative samples from a diverse set of optical water types [2,^4^,5]. The dataset described in this article [6] provides a diverse set of bio-optical data collected from Australian inland waterbodies between 2013 and 2021.

The dataset described in this article contains ten datafiles. Each datafile contains observations for a specific modality of bio-optical measurement. Measurements from each modality can be merged using the *observation_id* column, which provides a unique identifier for each set of observations.

**Fig. 1:**
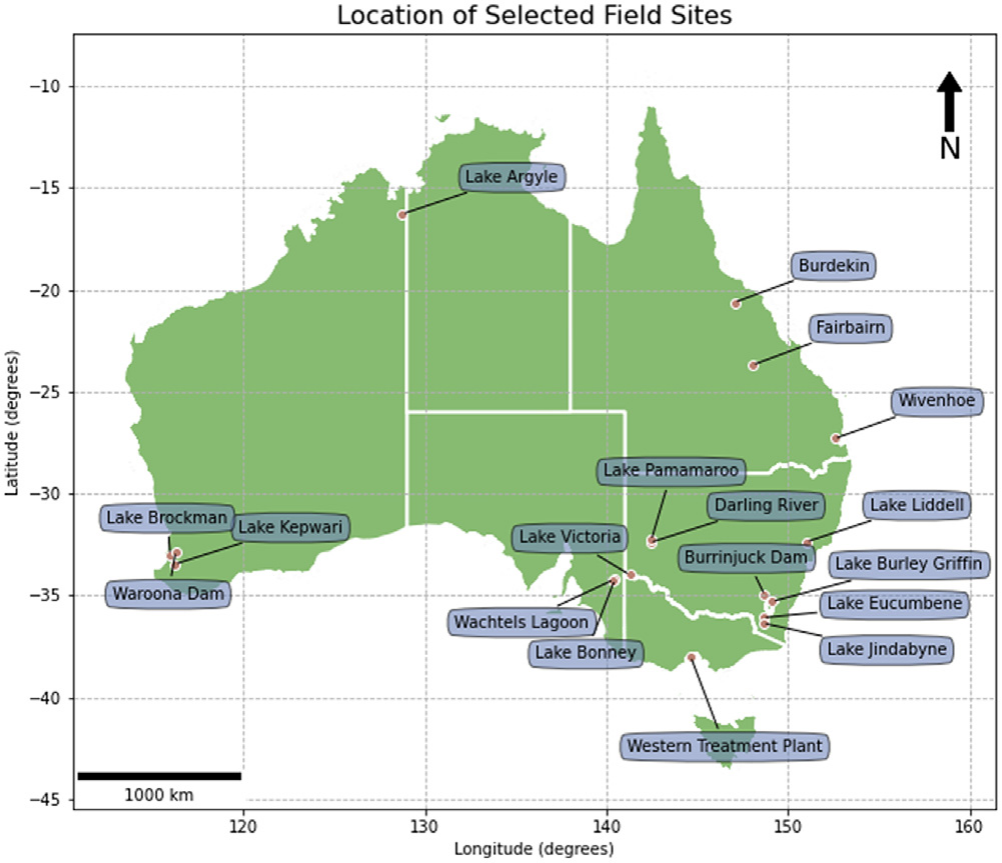
Locations of a selection of waterbodies.

The *observation* data file contains metadata and site observations. This file defines the location, date, time, and name of waterbody for each observation set. Data on cloud cover, water conditions, temperature, Secchi depth and colour are also provided. Fig. 1 shows the locations of selected waterbodies described by this dataset. Column definitions of the *observation* data file are provided in Table 1.

**Table 1:** Data description of observations metadata file.

Column name	Unit	Description
observation_id	ID	Unique identifier for each measurement set
site	ID	Unique identifier for each field site
datetime_utc	date time	date and time measurement acquisition commenced in UTC. Datetime displayed as YYYY-MM-DD HH:MM
waterbody	string	Name of waterbody
time_arrived_local	date time	Local date time measurement acquisition commenced. displayed as YYYY-MM-DD HH:MM
local_time_zone	string	Designation of local time zone
lat	float	Latitude in decimal degrees obtained with EPSG:4326
lon	float	Longitude in decimal degrees obtained with EPSG:4326
water_temp_degC	float	Surface temperature of the water in degrees Celsius
cloud_frac_pc	int	Percent fraction of cloud cover
water_colour	string	Short description of water colour
water_cond	string	Short description of water surface conditions
wind_speed_kn	float	Wind speed in knots
wind_direction	string	Cardinal wind direction
weather	string	Short description of weather conditions
secchi_depth_m	float	Secchi disc depth in meters
water_depth_m	float	Total water depth in meters
turbidity_ntu	float	Turbidity in Nephelometric Turbidity Unit (NTU)

**Fig. 2:**
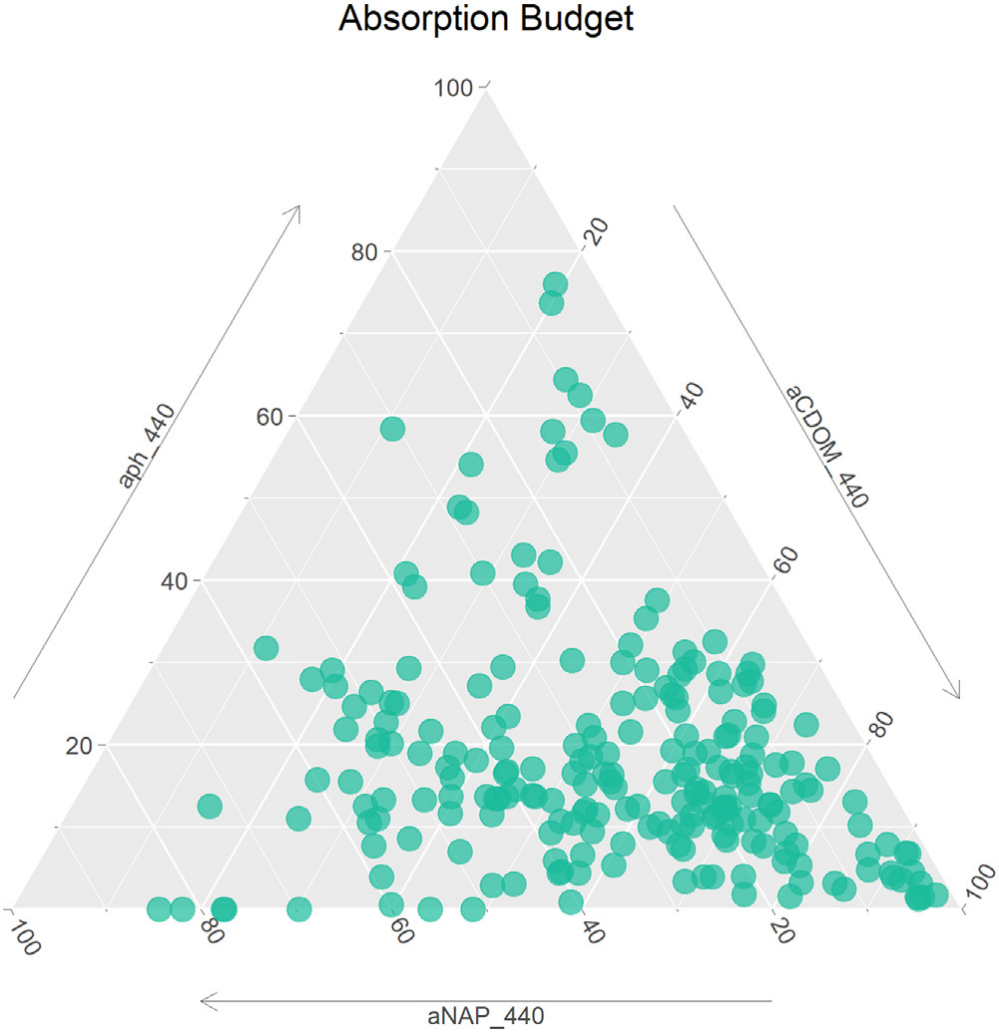
Absorption budget at 400 nm for samples in the absorption_fitted datafile.

Absorption coefficients and related parameters are presented in three data files, absorption_unfitted, absorption_fitted and absorption_slopes. Of these the unfitted datafile contains data obtained through direct measurement, while the absorption_fitted and absorption_slopes datafiles are derived from the unfitted data as described below (see 2.1). A total of 307 absorption measurements are available. The distribution of the absorption budget at 440 nm (Fig. 2) indicates that non-algal particles (NAP) and coloured dissolved organic matter (CDOM) are the primary absorption components for most observations in the dataset, with phytoplankton absorption (aph) dominating in comparatively few observations.

**Fig. 3:**
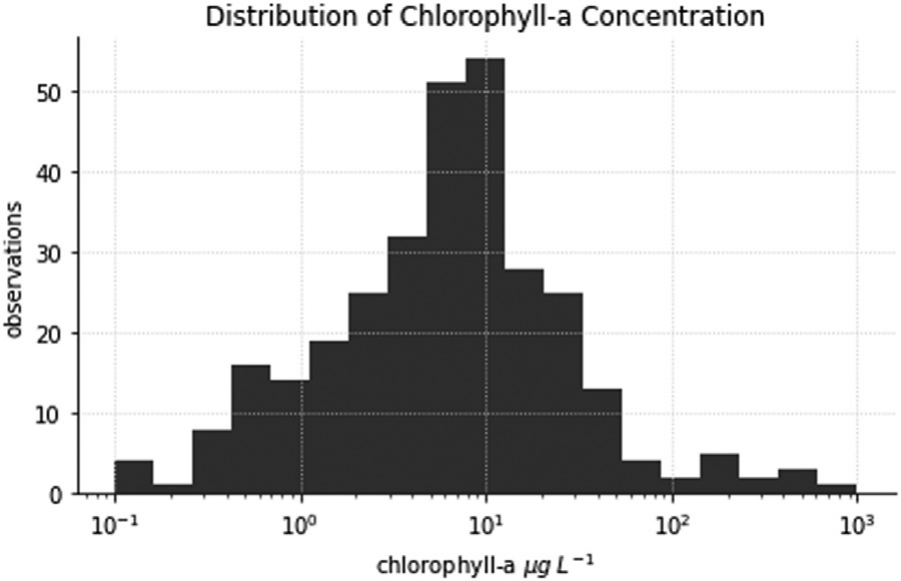
Distribution of chlorophyll-a concentrations.

The *pigments* datafile contains three hundred and eight observations of 28 algal pigments obtained using the HPLC method described in Clementson [1]. Chlorophyll-a (chla) concentrations show an approximately log-normal distribution in the dataset (Fig. 3). The mean and median chla concentrations for the dataset are 22.2 and 7.0 µg L^–1^, respectively.

The *org_c* datafile contains 174 observations of total and dissolved organic carbon. Concentrations of total organic carbon (TOC) range from 1.1 to 50 mg L^–1^ with dissolved organic carbon being the dominant form in the majority of samples.

The *tss* data file contains 309 measurements of total suspended solids (TSS). Observations of TSS range from 0.35 to 1786 mg L^–1^. The contribution of inorganic and organic particles to TSS is evenly distributed with 51% of samples comprised primarily of inorganic particles.

Radiometric measurements are presented in two datafiles, *radiometry* and *kd*. One-hundred and twenty remote sensing reflectance (Rrs) observations are provided in the *radiometry* datafile while seventy-four observations of the irradiance attenuation coefficient (K_d_) are provided in the *kd* data file. All radiometric data are provided in the spectral range of 350–900 nm at 1 nm resolution.

Two hundred and five particulate backscattering (b_bp_) measurements are available in the *bb_surface* datafile. Particulate backscattering values at 555nm predominate in the range 0.007–1 m^–1^ but values up to 4.76 m^–1^ were measured (Fig. 5). Observations at the higher end of this range were obtained through serial dilution of surface samples (see methods). The spectral slopes (ϒ_bbp_ Eq. (12)) of particulate backscattering are approximately normally distributed (Fig. 5). Some negative values of the spectral slope were obtained in low scattering waters and some waters with high concentrations of algal biomass.

**Fig. 4:**
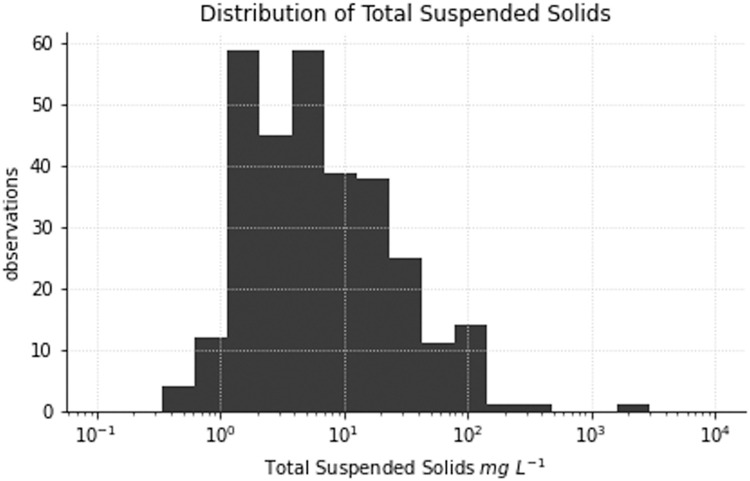
Distribution of total suspended solids (TSS).

**Fig. 5:**
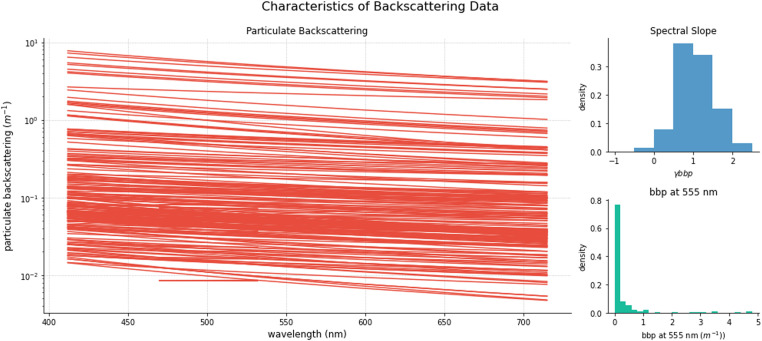
Characteristics of backscattering data, left particulate backscattering spectra, top right: distribution of particulate backscattering spectral slope, bottom right: particulate backscattering at 555 nm. Note backscattering spectra plotted using Eq. (12).

## 2 Experimental Design, Materials and Methods

### 2.1 Absorption Measurements

The *absorption_unfitted* datafile contains absorption coefficients for coloured dissolved organic matter (aCDOM), particulate absorption (ap) and detrital non-algal particulates (ad).

CDOM absorption coefficients were measured from surface (10–20 cm depth) water samples collected in 1L acid washed Schott bottles using the method described in Clementson et al. [7]. 80 mL of water was vacuum filtered using a 0.2 µm Whatman Anodisc filter to separate particulate matter. Filtered water samples were covered with aluminium foil to prevent light degradation and preserved 0.5 mL of a 10% *w/v* solution of sodium azide (NaN_3_). Samples were stored chilled and filtered within 24 h of collection and stored on ice for transport to the laboratory for analysis.

Particulate absorption samples were collected as for CDOM, using a clean 5 L polyethylene container. Samples were stored on ice and filtered using Whatman GF/F filters. Filters were stored flat in cryo-cages, covered in aluminium foil, and stored in liquid nitrogen until analysis.

CDOM samples were gradually warmed to room temperature and transferred to a 10 cm quartz cell with their absorbance spectra measured from 210 to 900 nm using a Cintra 404 UV/vis dual-beam spectrophotometer and Milli-Q water (Millipore) as a reference. Absorption coefficients were calculated using Eq. (1) where A(λ) is the absorbance normalised to zero at 680 nm and *l* is the cell pathlength in meters [7].

(1) $$a{\left(\lambda \right)}_{CDOM}\;=\;2.3\left[A\left(\lambda \right)/l\right]$$

(2) $$a\left(\lambda \right)\;=\;a_{350}exp\left[-S\left(\lambda -350\right)\right]\;+\;b$$

Absorbance scans for total particulate and non-algal particulate matter were obtained using a Cintra 404 UV/vis dual-beam spectrophotometer equipped with integrating sphere. Particulate optical density spectra were obtained using glass plates to hold sample and blank filters against an integrating sphere. Blank filters, from the same batch as the sample filters, were wetted with small volumes of filters sample and used as a reference. Optical density scans were made from 210 to 900 nm with a spectral resolution of 0.85 nm. A methanol extraction was then used to remove phytoplankton from the filter [8]. The filters were re-scanned to obtain non-algal particulate (NAP) optical density spectra.

Absorbance scans were corrected for path length amplification using the coefficients from Mitchel [9] to obtain absorption coefficients. Measured data are provided in the *absorption_unfitted* datafile.

Fitted and derived absorption values are provided in the *absorption_fitted* and *absorption_slopes* datafiles, respectively. The fitted datafile contains absorption coefficients for phytoplankton (a_ph_), CDOM (a_cdom_) and non-algal particulate matter (a_nap_).

Fitted CDOM and NAP absorption data are obtained by fitting measured absorption coefficients to Eq. (2) where a_350_ is the absorption coefficient at 350 nm, *S* is the spectral slope, λ is the wavelength in nm and b is an offset used in baseline correction. Fitted data remove imperfections in the measured spectra such as residual phytoplankton absorption in the NAP measurement. Phytoplankton absorption spectra are calculated as the difference between particulate absorption and detrital absorption from the *absorption_unfitted*. datafile The *S* parameter from Eq. (2) is included in the *absorption_slopes* datafile.

Total particulate absorption spectra were smoothed using a 10 nm running boxcar filter and the fitted NAP spectra subtracted to obtain phytoplankton absorption.

Quality control flags are provided for the absorption data to indicate data with missing spectral components, derived values, unusual shapes and suspected sampling or laboratory errors. Quality flags are provided to enable users to evaluate the data for their purposes.

Quality flag 1 indicates that some data have been provided with nominal values as the absorption component was below the detection limit. In these cases, a nominal absorption spectrum of 10^–6^ m^–1^ at every wavelength was included to enable users to derive specific inherent optical properties from the data. On one field trip CDOM absorption spectra were negative at some sites. As the water body was small and relatively homogeneous non-negative CDOM spectra were averaged to provide estimated absorption coefficients. Tables 2 and 3 show the column definitions for the *absorption_unfitted* and *absorption_fitted* datafiles. Table 4 shows the quality flags and their definitions.

**Table 2:** Data description of the unfitted absorption dataset.

Column name	Unit	Description
observation_id	ID	Unique identifier for each measurement set
aCDOM	m^–1^	Absorption coefficient for coloured dissolved organic matter
ad	m^–1^	Absorption coefficient for non-algal particles
ap	m^–1^	Absorption coefficient for particles
abs_wls	nm	wavelengths of absorption coefficients
qflag_aCDOM_uf	integer	quality control flag for CDOM absorption coefficients
qflag_ad_uf	integer	quality control flag for non-algal particles absorption coefficients
qflag_ap_uf	integer	quality control flag for particulate absorption coefficients
comment	string	description of quality control issues

**Table 3:** Data description of the fitted absorption dataset.

Column name	Unit	Description
observation_id	ID	Unique identifier for each measurement set
aCDOM	m^–1^	Absorption coefficient for coloured dissolved organic matter
ad	m^–1^	Absorption coefficient for non-algal particles
aph	m^–1^	Absorption coefficient for phytoplankton
abs_wls	nm	Wavelengths of absorption coefficients
qflag_aCDOM_ft	integer	Quality control flag for CDOM absorption coefficients
qflag_ad_ft	integer	Quality control flag for non-algal particles absorption coefficients
qflag_aph_ft	integer	Quality control flag for phytoplankton absorption coefficients
comment	string	Description of quality control issues

**Table 4:** Quality control flags for absorption spectra.

Quality flag	Definition
0	No quality issues
1	derived value from averaging of similar sites on the same field trip
2	derived value from below detection limit
3	missing laboratory/field error/unable to obtain
4	treat with caution unusual value

### 2.2 Pigments

The HPLC method described in Clementson [1] was used to obtain the concentration of 28 algal pigments including chlorophyll-a. Samples for pigment analysis were collected in clean 5L HDPE containers at a depth of 10–20 cm, cooled for storage and filtered within 24 h. Vacuum filtration was performed using Whatman® glass microfiber filters, Grade GF/F, 0.7 μm until the filter paper was coloured by the sample. Typical filtration volumes ranged from 0.5 to 2 L depending on the concentration of chlorophyll and non-algal particulates present in the sample. Filter papers were folded in half and stored in liquid nitrogen until analysis. Prior to analysis filter papers are thawed and cut into 3–4 pieces. Pigments are extracted by centrifuge with 3 mL of 100% acetone. The samples are then chilled in an ice bath and then kept in the dark at 4°C for 15 h. Water is then added to the extraction mixture to make a 90:10 acetone:water solution and the sample is sonicated. The sample is centrifuged at 2500 rpm for five minutes at -2°C to separate the extract from the filter paper. The filtrate is then passed through a 0.2 µm Teflon syringe filter into a 2 mL amber HPLC vial. An auto sampler chilled to 4°C is used to apply an aqueous tetrabutyl ammonium acetate (TbAA) methanol solution immediately prior to sample injection. Following injection pigments are separated on a Zorbax Eclipse XDB-C_8_ stainless steel 150×4.6 mm chromatographic column and gradient eluted using a TbAA:methanol solvent [1]. Separated pigments are detected using a PDA detector and identified against standard spectra.

Pigments data are collected in the *pigments* datafile. All pigments are listed with concentration units of µg L^–1^. The ‘tot_mv_chl_a’ column contains total chlorophyll-a concentrations consisting of the sum allomeric and epimeric forms of chlorophyll-*a*.

### 2.3 Organic Carbon

Total and dissolved organic carbon (TOC and DOC) are provided in a single data file, *org_c*. Organic carbon samples are collected as for the CDOM samples above. DOC samples are filtered in the same manner as CDOM, and the filtrate acidified with 0.5 mL of 50% H_2_SO_4_ solution and stored in acid washed glass containers wrapped in aluminium foil at 4°C for transport to the laboratory. Unfiltered TOC samples are treated identically to DOC samples. TOC and DOC are measured using a Shimadzu Total Organic Carbon Analyser (TOC-V_CHS/CSN_+ TNM-1). Samples are acidified and purged with CO_2_-free air, then combusted at 720°C and measured by a nondispersive infrared detector. Table 5 provides column definitions for the organic carbon datafile.

**Table 5:** Data description of organic carbon dataset.

Column name	unit	description
observation_id	ID	Unique identifier for each measurement set
DOC	mg/L	Dissolved organic carbon in mg/L
TOC	mg/L	Total organic carbon in mg/L

### 2.4 Total Suspended Solids

Total Suspended Solids (also referred to as Total Suspended Matter) (TSS) data are provided in a single datafile, *tss* (Fig. 4). TSS samples were collected from the upper 10 to 20 cm of the waterbody in 5 L high-density polyethylene containers. Triplicate TSS samples were subsamples from the 5 L container. A known volume of each subsample was filtered through a pre-ashed at 450°C and pre-weighed filter by vacuum filtration using Glass fibre filters (0.7 µm) prepared after Tilstone, et al. [10]. Following filtration, the filters were stored in the cool and dark while being transported to the laboratory for analysis. The filters were dried at 75°C with an initial drying period of 24 h. TSS is taken as the mass difference between the original filter weight minus the final weight divided by the volume of sample used. The contribution of organic and inorganic material to TSS was determined by the mass difference in TSS following combustion at 450°C for 3 h. TSS data are reported as averages and standard deviations made from triplicate measurements. Column definitions for the TSS datafile are given in Table 6.

**Table 6:** Data description of TSS dataset.

Column name	unit	description
observation_id	ID	Unique identifier for each measurement set
tss	mg/L	Average total suspended solids from three replicates
tss_inorganic	mg/L	Average inorganic suspended solids from three replicates
tss_organic	mg/L	Average organic suspended solids from three replicates
tss_std	mg/L	Standard deviation of total suspended solids from three replicates
tss_inorganic_std	mg/L	Standard deviation of inorganic suspended solids from three replicates
tss_organic_std	mg/L	Standard deviation of organic suspended solids from three replicates

### 2.5 Radiometry

Radiometry data are provided in the *radiometry* and *Kd* data files. The *radiometry* data file provides measurements of the remote sensing reflectance (R_rs_) (Eq. (3)) evaluated just above the water air interface where E_d_^0+^(λ) is the spectral planar downwelling irradiance at the water surface and L_w_(λ,θ,φ) is the water leaving radiance at wavelength λ, at nadir viewing angle θ and azimuth angle φ just above the water surface as defined in Ruddick et al. [11]. For clarity the spectral component of radiometric quantities (λ) is omitted for equations following Eq. (3).

The *radiometry* data file identifies two methods of data collection, ‘interface’ and ‘kutser’. Each method is described separately below. All radiometric measurements are obtained using three Trios RAMSES sensors — a RAMSES ACC-2 VIS planar irradiance sensor (E_s_), a RAMSES ACC-2 diffuse irradiance sensor (E_d_) and a RAMSES ARC VIS radiance sensor.

Interface measurements follow the protocol for the ‘single-depth approach’ described in Zibordi and Talone [12].

**Fig. 6:**
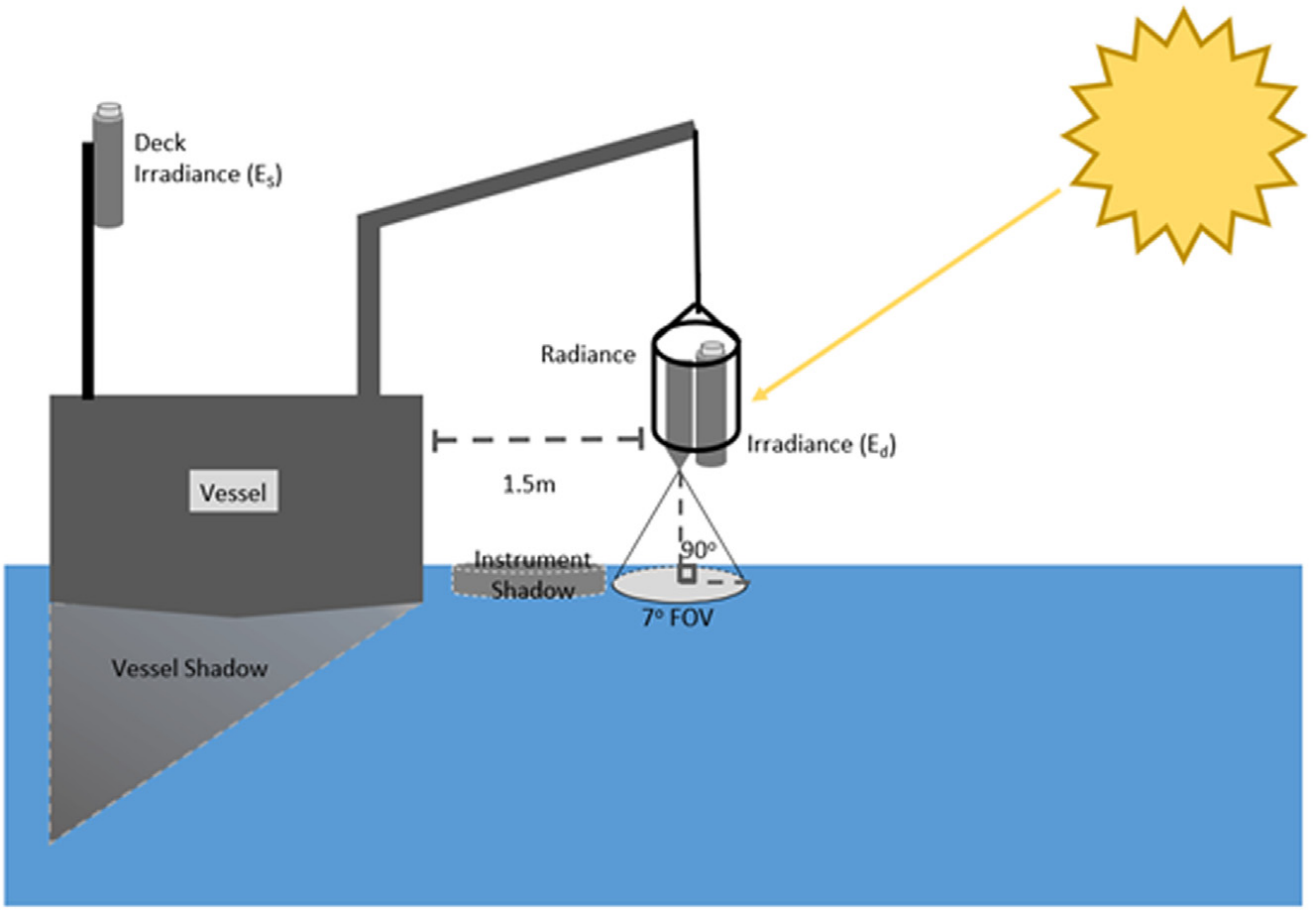
Radiometry measurement geometry.

**Fig. 7:**
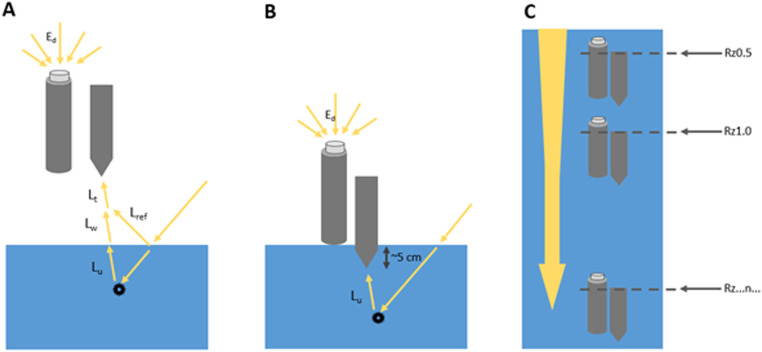
radiometric measurement stages — (A) above surface, (B) interface and (C) profile. At the above surface stage, L_T_ is the total radiance reaching the sensor, this quantity is composed of reflected light (L_ref_) and the water leaving radiance (L_w_). L_w_ consists of the upward radiance (L_u_) transmitted through the water surface.

Radiometers are positioned according to Fig. 6 to minimise shading from the instrument and vessel. Radiometric measurements are conducted in three phases shown in Fig. 7. The phases include above surface measurements (Fig. 7A), an interface stage (Fig. 7B) and a profiling stage (Fig. 7C). Radiometric measurements are made over a period of 20–30 minutes, during which time the light field is subject to change. To ensure all data are comparable, data from each measurement stage are normalised to the deck E_s_ sensor [13] as shown in Eq. (4), where m_i_ is the measurement made at time *i* with E_s_(t^i^) and E_s_(t^0^) as the deck E_s_ measurements at time *i* and time = 0 respectively.

(3) $$R_{rs}\left(\lambda ,\;\theta ,\;\phi \right)\;=\;\frac{L_{w}\left(\lambda ,\;\theta ,\;\phi \right)}{E_{d}^{0+}\left(\lambda \right)}$$

(4) $$m_{norm}\left(t^{i}\right)\;=m_{i}\left(t^{i}\right)\cdot \;\frac{\;E_{s}\left(t^{0}\right)}{E_{s}\left(t^{i}\right)}$$

The above surface phase (Fig. 7A) involved radiance and irradiance measurements made at approximately 40 cm above the water surface.

The interface phase (Fig. 7B) measures the upwelling radiance (L_u(z)_) 5–10 cm below the water surface and calculates R_rs_ according to the single depth approach (SDA) described in [12]. L_u(z)_ is corrected for vertical attenuation between the measurement depth (z_1_) and the water/air interface (L_u(0-)_) according to Eq. (5). L_u_(z_1_,t_1_) is the radiance measurement made at depth z_1_ and at time t_1_ shown in Fig. 7B, with K_Lu_ being derived from the profiling phase (Fig. 7C). K_Lu_ is determined by examining the vertical attenuation of radiance measurements over the depth range z_1_-z_2_ measured during the profile phase (Fig. 7C). Typically, 5–10 measurements of the upwelling radiance are made during the profiling stage. L_u_(z_2_,t_2_) is selected from these measurements in order to minimise instrument noise but maximise the depth over which K_Lu_ is calculated. Typically L_u_(z_2_,t_2_) is taken as the second deepest measurement in the profile.

L_u_(0-) is then converted to the water leaving radiance (L_w_0+) by accounting for the spectral transmittance across the water-air interface using Eq. (7) [11] where T_F_ is the Fresnel transmittance of radiance from water to air and n_w_ is the refractive index of water and L_u_(0-) is the upwelling irradiance just below the water surface.

(5) $$L_{u}\left(0-\right)\;=\;L_{u}\left(z_{1},\;t_{1}\right)exp\left(K_{Lu}\;z_{1}\right)$$

(6) $$K_{Lu}\;=\;\frac{1}{z_{2}-z_{1}}ln\left[\frac{L_{u}\left(z_{1},\;t_{1}\right)}{L_{u}\left(z_{2},\;t_{2}\right)}\right]$$

(7) $$L_{w}\left(\lambda \right)\;=\;\frac{T_{F}}{n_{w}^{2}}L_{u}\left(0^{-},\;\lambda \right)$$

For flat seawater a spectrally independent value of 0.543 [14] for $\frac{T_{F}}{n_{w}^{2}}$ is regularly used in the literature. In the current dataset the spectrally dependant transmittance is calculated after the procedure described in [15].

Following correction for transmittance across the water-air interface, R_rs_ is then calculated as in Eq. (3). To avoid interference from the vessel, the E_d_ obtained during above surface phase (Fig. 7A) is used in this calculation. This is the quantity reported with the ‘interface’ flags in the *radiometry* datafile.

At several locations the single-depth approach described above were not able to be made due to the contaminated nature of the waterbody or strong vertical attenuation generating noise in the radiance sensor at shallow depths (<0.5 m). At these sites the protocol described by Kutser et al. [16] was adopted. This method uses the measurement geometry shown in Fig. 7A. Kutser measurements are obtained by fitting a power law function to the ratio of L_T_/E_d_ over the spectral range 350–380 and 890–900 nm. The function is then applied to the full spectral range and subtracted from L_t_/E_d_ to give an estimate R_rs_ referred to as R_rs(k)_. This quantity is reported in the *radiometry* datafile identified by the ‘kutser’ flag in the measurement column.

K_d_ measurements are obtained by sequentially lowering the irradiance (E_d_) sensor as shown in Fig. 7C. Measurements of downwelling irradiance E_d(z)_ are made at depths (z_i_) to obtain the spectral diffuse attenuation coefficient (k_d_). Irradiance measurements (E_d_) are fit to Eq. (8), where E_d_(0^–^, λ) is the irradiance at wavelength λ measured just below the water surface and z is depth in meters.

(8) $$E_{d}\left(z,\lambda \right)\;=\;E_{d}\left(0^{-},\;\lambda \right)\cdot \;exp\left(-\int_{0}^{z}k_{d}\left(z,\lambda \right)\cdot dz\right)$$

The number and sequence of depth intervals obtained were determined based on the rate that the optical signal diminished with depth and depth of the waterbody. Surface waves are known to introduce optical shading and amplification effects in E_d_ and to a lesser extent in L_u_ profiles [17]. To reduce this effect 10–20 measurements were made at each depth interval, from which a trimmed average was taken using the middle 50% of measurements. The middle 50% of measurements were determined through numerical integration of the spectrum to a zero baseline.

Radiometric measurements are provided in two data files described in Tables 7 and 8 below.

**Table 7:** Data description for *radiometry* dataset.

Column Name	Unit	Description
observation_id	ID	Unique identifier for each measurement set.
datetime_utc	Date-time	Date-time for start of measurement. Format yyyy-mm-dd hh:mm:ss:f in universal time coordinated time scale.
method	categorical	Confirms interface or kutser methods.
Rrs	sr^–1^	Remote sensing reflectance (Eq. (3))
wls	nm	Wavelengths radiometric measurement.

**Table 8:** Data description for k_d_ dataset.

Column Name	Unit	Description
observation_id	ID	Unique identifier for each measurement set
Ed_zero	mW m^–2^	E_d_ 0^–^, irradiance at 0m below the water surface extrapolated from fitting data to Eq. (8).
Kd	m^–1^	Diffuse attenuation coefficient extrapolated from fitting data to Eq. (8).
Kd_std_error	m^–1^	Standard error in K_d_.
Kd_wls	nm	Wavelengths for K_d_ in nanometres.

### 2.6 Backscattering

Backscattering data were collected using a Seabird ECO BB9 backscatter meter [18] and in some cases an ECO triplet (bb2) [19]. The data are provided in the *bb_*surface datafile. The bb9 and bb2 provide data of the total volume scattering coefficient (β_T_(124⁰, λ)) where θ is the receiving angle of the instrument (124⁰) and λ is the wavelength. Wavelengths available for the BB2 are 470, 532 nm while the BB9 measures are 412, 440, 488, 510, 532, 595, 650, 676, 715 nm.

Three methods designated, bucket, profile, and dilution, were used to obtain backscattering data. The bucket method involved sampling approximately 9L of water from the surface water. The water sample was then transfer to an opaque black vessel that was filled to enable the BB9 sensors to be submerged. Measurements were made over a two-minute period from which an average was taken for further processing. The vessel was covered during measurement to prevent the incursion of ambient light.

The profiling method involved attaching the BB9 to a metal cage suspended from a winch. A pressure sensor was used to determine depth as the cage was lowered through the water column. Back scattering measurements from the upper 1.5 m were averaged for use in further processing.

The BB9 was developed for use in oceanic and coastal waters and is subject to saturation in highly scattering inland waters. To provide comprehensive coverage of Australian bio-optical conditions the authors used a serial dilution technique to estimate the backscattering properties of highly turbid waterbodies. This technique involved obtaining ∼20 L of sample water obtained from the first 50 cm of surface waters. Seven litres of water were transferred to an opaque black bucket vessel and the sample was then progressively diluted using 18 MΩ MilliQ water until all wavelengths were unsaturated. The sample water was then gently stirred to ensure particles remained suspended and a two-minute measurement made. This process was repeated 4–5 times to obtain measurements over a wide concentration range. Linear regression was then used to estimate the particulate backscattering at full concentration. Further details of this method and validation analysis are being prepared as part of a forthcoming publication. All observations are tagged to enable users to filter or select data based the measurement method and their requirements.

β_T_(124⁰, λ) are corrected for absorption using Eq. (9), where L_p_ is the optical pathlength in meters and a_T_ is the total absorption (including water). The manufacturer recommends using 0.0391m for L_p_, however Monte Carlo simulations in highly absorbing waters have indicated a shorter path length of 0.01635 is a more appropriate value [20]. This value has been adopted for this dataset. All uncorrected values are provided in the dataset for the convenience of users. Total absorption (a_T_) was calculated as the sum of CDOM, phytoplankton and NAP absorption from the *absorption_fitted* datafile. Absorption of water was obtained from Buiteveld et al. [21].

β_corrected_ (124⁰, λ) coefficients are converted to particulate volume scattering coefficients (β_p_ (124⁰, λ) by subtracting the volume scattering function for water (β_water_(124⁰, λ)) after Eq. (10), where λ is the wavelength in nanometres and PSU is the salinity in practical salinity units. The particulate back scattering coefficients (b_bp_(λ)) are then estimated using Eq. (11).

(9) $$\beta_{\text{corrected}}\left(124^{0},\lambda \right)=\beta_{\text{measured}}\left(124^{0},\lambda \right)\cdot exp\left(L_{p}\cdot a_{T}\right)\#$$

(10) $$\beta_{water}\left(124^{0},\lambda \right)=1.38\cdot {\left(\frac{\lambda }{500}\right)}^{-4.32}\cdot \left(1\;+\;0.3\;\frac{PSU}{37}\right)\cdot 10^{-4}\cdot \left(\frac{1+cos^{2}124\cdot \left(1-0.09\right)}{1\;+\;0.09}\right)$$

(11) $$b_{bp}\left(\lambda \right)=2\pi \cdot 1.1\cdot \beta_{p}\left(124^{0},\lambda \right)$$

The particulate backscattering spectral slope (ϒ_bbp_) and backscattering at 555 nm (bbp_555) are calculated by fitting the bbp data to Eq. (12) using least squares regression on the natural log transformed data. Blue wavelengths are known to periodically deviate from the relationship described in Eq. (12). To account for this all combinations of seven of the nine wavelengths are fit to Eq. (12) with the combination that produces the smallest residuals being retained. Colum definitions for the *bb_surface* datafile are shown in Table 9.

(12) $$bbp\left(\lambda \right)\;=\;bbp_{555}{\left(\frac{555}{\lambda }\right)}^{\gamma }$$

**Table 9:** Data description for the bb_surface datafile.

Column Name	Unit	Description
observation_id	ID	Unique identifier for each measurement set
method	categorical	Method used, one of: • surface_bb2 - measurement made at surface using bb2 • dilution - obtained using serial dilutions using bb9 • bucket - measurement made in bucket using surface water sample • bb9_*in situ* - obtained from *in situ* measurement using bb9
beta_raw	m^–1^ sr^–1^	Volume scattering function at 124 degrees at bbwls wavelengths.
beta_cor	m^–1^ sr^–1^	Absorption corrected volume scattering function at 124 degrees at bbwls wavelengths.
bbp	m^–1^	Particulate backscattering coefficients at bbwls wavelengths.
bbwls	nm	Wavelengths of the backscattering meter in nanometres.
gamma_bbp	-	Particulate backscattering spectral slope obtained from the 7 wavelengths that best fit Eq. (12)
bbp_555	m^–1^	Particulate backscattering at 555 nm

## Ethics Statements

This work did not include human subjects, animal experimentation or data collection from social media platforms.

## CRediT authorship contribution statement

**Nathan Drayson:** Writing – original draft, Formal analysis, Investigation, Data curation, Software, Visualization. **Janet Anstee:** Conceptualization, Methodology, Project administration, Funding acquisition, Investigation, Supervision. **Hannelie Botha:** Conceptualization, Methodology, Investigation, Supervision. **Gemma Kerrisk:** Investigation, Resources. **Phillip Ford:** Investigation, Supervision. **Bozena Wojtasiewicz:** Investigation, Formal analysis, Methodology. **Lesley Clementson:** Investigation, Formal analysis, Methodology. **James McLaughlin:** Investigation. **Marlee Hutton:** .

## Declaration of Competing Interest

The authors declare that they have no known competing financial interests or personal relationships that could have appeared to influence the work reported in this paper.

## Data Availability

Bio-optical data for Australian Inland Waters v.1 (Original data) (CSIRO Data Access Portal).
